# Effect of cryomilling time on microstructure evolution and hardness of cryomilled AZ31 powders

**DOI:** 10.1016/j.matchar.2021.111311

**Published:** 2021-08

**Authors:** Dikai Guan, Junheng Gao, W. Mark Rainforth

**Affiliations:** Department of Materials Science and Engineering, University of Sheffield, Sheffield S1 3JD, UK

**Keywords:** Cryomilling, AZ31, TEM, XRD, Hardness, Grain refinement

## Abstract

The synthesis of nanostructured AZ31 powder by cryomilling was studied in this paper. The microstructural evolution during cryomilling, including the changes of particle morphology and internal grain size, was characterized via optical microscopy, SEM, TEM and XRD. Observations during the cryomilling produced four main findings. Firstly, cryomilling can refine the grains of AZ31 particles down to 100 nm after around 1 h milling and the minimum average grain size of about 30 nm was reached when the cryomilling time was extended to 6 h or longer. Secondly, cold welding played a dominant role in the early stage of cryomilling, while fracture took place in the late stage and surpassed cold welding. The former led to a particle size increase while the latter decreased the particle size. The minimum average particle size after 6 h cryomilling was approximately 26 μm. Thirdly, a few particles were agglomerated with other particles and could not be processed by cryomilling due to cold welding. Finally, after cryomilling 6 h and longer times, the hardness reached 162 HV which was much higher than other values reported in AZ31 alloy studies.

## Highlights

• Cryomilling refined the grains down to 100 nm after around 1 h milling.
• The minimum average grain size of about 30 nm was reached after cryomilling 6 h.
• The hardness reached 162 HV, much higher than other values reported in AZ31 studies.

## 1 Introduction

Recently, cryomilling has attracted considerable attention because of its ability to produce nanocrystalline and other non-equilibrium structures and bulk materials followed by appropriate consolidation quickly compared to conventional high energy ball milling. For example, Khan et al. recently reported the grain sizes of Mg-xAl (x = 0, 5, 10 and 20 wt%) can be refined below 100 nm after high energy ball milling for 100 h [1], but our previous work showed that the nanostructured Mg particles can be obtained only after cryomilling for 6 h [2,3]. One of the early literature to present the application of cryomilling was a Al–Al_2_O_3_ composite [4]. Subsequently, cryomilling has been widely used to produce nanostructured materials such as Ni [5,6], Al [[7], [8], [9], [10], [11], [12]], Fe [13], Zn [14], and Ti [15,16] alloys. Considering the advantages of cryomilling and the process obtained in other alloys system, cryomilling has been used to fabricate and successfully synthesize nanostructured bulk magnesium-based alloys [[17], [18], [19], [20], [21], [22]]. The reduction in grain size in Mg and its alloys to the nanoscale (NS) by conventional routes is prevented by dynamic recovery and recrystallization [23,24]. In contrast, cryomilling provides a facile method to produce nanostructured (NS) materials.

For commercially pure Mg and alloys such as AZ31 with low alloying content, it is a challenge to produce even a UFG microstructure, let alone a NS microstructure using conventional processing, due to the rapid growth kinetics of the single-phase grains. In the past years, the combination of cryomilling and spark plasma sintering (SPS) was successfully used to fabricate NS pure Mg, AZ31, Mg—10Al, Mg—30Al and AZ80 alloys [2,3,18,19,21,[25], [26], [27], [28]]. However, the grain refinement evolution of AZ31 particles during cryomilling process has not been reported in detail before.

Therefore, the primary two objectives of this study are as follows. First, to find out how effective is the cryomilling for grain refinement of Mg AZ31 particles? Second, how long does it take for the AZ31 particles to reach the minimum grain size when processed by the cryomilling?

## 2 Experimental

### 2.1 Mg alloy powder

The as-received powder was 200 mesh (−75 μm) helium gas atomized AZ31 powder provided by Magnesium Elektron Ltd., USA. Particles smaller than 38 μm were removed by a sieve (400 mesh) before cryomilling. Therefore, the particle size of the precursor powder was distributed in the range of 38–75 μm. The particle size distribution of this precursor powder and the internal average grain size of around 2 μm were reported in our previous work [3].

### 2.2 Cryomilling

A cryomill with an integrated cooling system (Retsch, Germany) was employed in this work. The grinding jar was continually cooled with liquid nitrogen during the whole grinding process. Liquid nitrogen circulated through the system and was continually replenished from an autofill system to keep the temperature at −196 °C and avoid direct contact with liquid nitrogen.

6 g of AZ31 powders and a stainless steel ø 25 mm grinding ball were loaded into the stainless steel 50 ml grinding jar in an argon atmosphere glovebox (MBRUAN, Germany). One cycle of the cryomilling process involved precooling, grinding and intermediate cooling. Detailed experimental parameters are shown in Table 1. The powders were cryomilled for various time intervals: from 15 min to 8 h, corresponding to 3 to 96 cycles.

**Table 1:** Cryomilling process parameters during one cycle.

Precooling		Grinding		Intermediate cooling	
Frequency(Hz)	Time(min)	Frequency(Hz)	Time(min)	Frequency(Hz)	Time(min)
5	18	22	5	5	3

### 2.3 X-Ray Diffraction (XRD)

A Siemens X-Ray Diffractometer D5000 using Cu Ka (λ = 0.15406 nm) radiation was employed to study the cryomilled powders. The diffractometer has a programmable divergence slit with a 0.02 rad Soller and a 1degree divergence slit on the Cu Kα x-ray source. The detector was set to read from 30° to 80° at 2.4 s/step with a step size of 0.02°. Fully annealed as-received AZ31 powder was used as a standard to subtract instrumental broadening. XRD peak profiles were fitted by a Pearson VII function, and full width at half maximum (FWHM) was used as a measure of peak broadening. The pure sample peak broadening B was calculated using.

$B=\sqrt{{B_{\mathrm{obs}}}^{2}-{B_{\mathrm{inst}}}^{2}}$, where *B*_*obs*_ is the observed peak broadening, and *B*_*inst*_ is the instrumental broadening [29].

### 2.4 Microstructure characterization

Optical microscopy was carried out on a Nikon (Eclipse LV150) microscope. Images were taken at various magnifications to study the microstructure and grain size. Samples were cold mounted and ground with SiC paper from 800 to 4000 grit. To minimize oxidation exposure to water, samples were polished with alcohol based diamond suspensions of 1 and 0.25 μm. Some samples were etched using an acetic-picral solution (4.2 g picric acid, 10 ml acetic acid, 70 ml ethanol and 10 ml water) for 1 s.

The sample preparation procedure for SEM was the same as that of optical microscopy, but etching was not necessary in some cases. A FEGSEM (Inspect F, FEI) was employed to investigate the morphology of powders.

For particle size analysis of cryomilled powder, the cryomilled Mg particles were distributed and pressed on a carbon stick on top of a SEM pin stub. Loose particles were removed by using compression air. At least 200 particles were measured using Nano Measure software to calculate the average particle size of all cryomilled powder.

TEM samples were prepared by grinding using a motar and pestle and suspended in isopropanol, followed by ultrasonic dispersion and then deposition onto a 200 mesh Cu grid with holey carbon film. A FEI Tecnai 20, operating at 200 kV, was used for conventional TEM characterization.

### 2.5 Chemical analysis

Chemical analysis was conducted by London & Scandinavian Metallurgical Co Limited, Sheffield, UK. Elemental analysis was requested and results reports were returned, for oxygen, nitrogen and iron, because these elements are expected to be the main impurity species in cryomilled powders during the cryomilling process.

### 2.6 Microhardness test

To obtain a flat surface for hardness test, the cold mounted powder samples were ground with 800, 1200, 2500, 4000 grade SiC papers and finally polished by 1 μm and 1/4 μm alcohol based diamond suspension. A Vickers hardness tester (Akashi Corporation Sagami Plant, model HM-101) was used to perform hardness tests with a load of 50 g and a dwell time of 15 s. At least 11 indents were collected for each sample to obtain accurate hardness results.

## 3 Results and discussion

### 3.1 Particles morphologies evolution during cryomilling

**Fig. 1:**
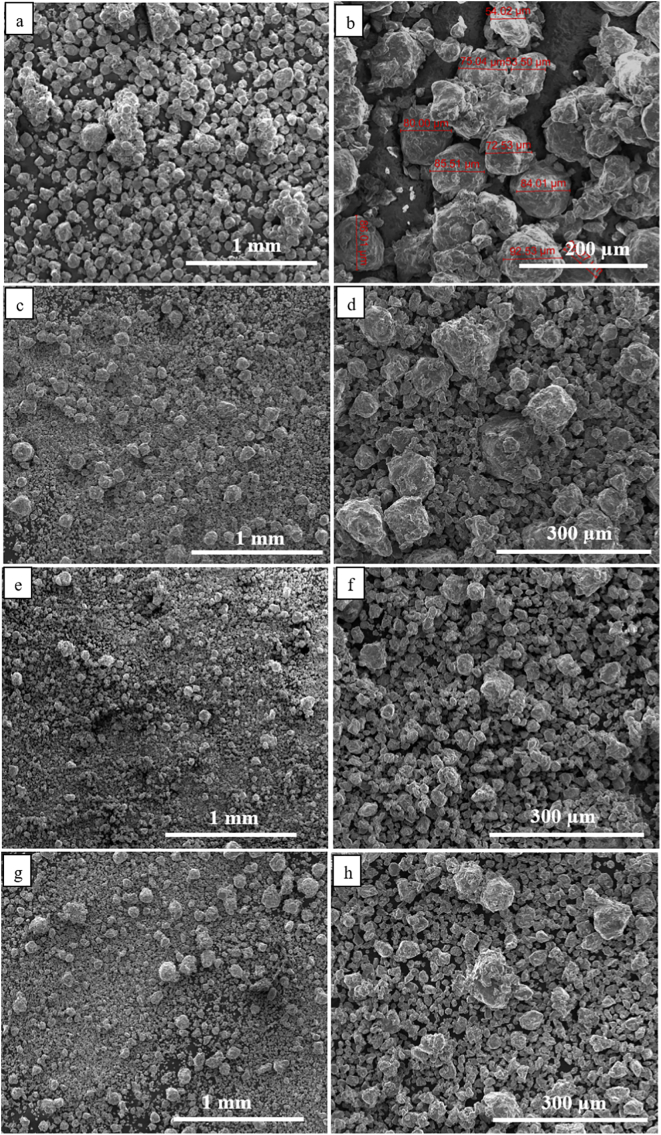
Typical secondary electron SEM images showing particles morphology of cryomilled powders after (a–b)1 h, (c–d) 5 h, (e–f) 6 h, (g–h) 8 h.

Fig. 1 shows the particle morphology after different cryomilling times. It should be noted that only 1 h, 5 h, 6 h and 8 h cryomilled powders were selected here which were regarded as sufficient to show the particle morphology evolution. As shown in Fig. 1(a, b), it can be clearly seen that most of the 1 h cryomilled particles were larger than the precursor powder particle size (38–75 μm) and some particles were also agglomerated. However, with increasing the cryomilling time to 5 h, approximately half particles were reduced in size due to the milling. After 6 h cryomilling, more coarse particles were crushed into fine particles with an average size of 26 μm. Finally, no significant change in particle size was observed after 8 h cryomilling compared to the 6 h cryomilled powders. Therefore, there was no point in extending the cryomilling time further. The evolution in particle morphology can be explained by cold welding and fracture occurring during the whole cryomilling process [16,[30], [31], [32]]. The grinding ball with a high shaking frequency plastically deforms the particles resulting in work hardening and fracture. The freshly fractured surface assists the deformed particle to weld together leading to increase particle size. However, with continued severe cold deformation, fracture plays a dominant role over cold welding and reduces the particles size. Finally, cold welding and fracture reach a balance and the particle size does not change. Although the particle size is not evidently changed, the internal structure of the particles is increasingly refined due to the continued high energy impact of grinding ball, which is discussed below.

### 3.2 Microhardness evolution during cryomilling

**Fig. 2:**
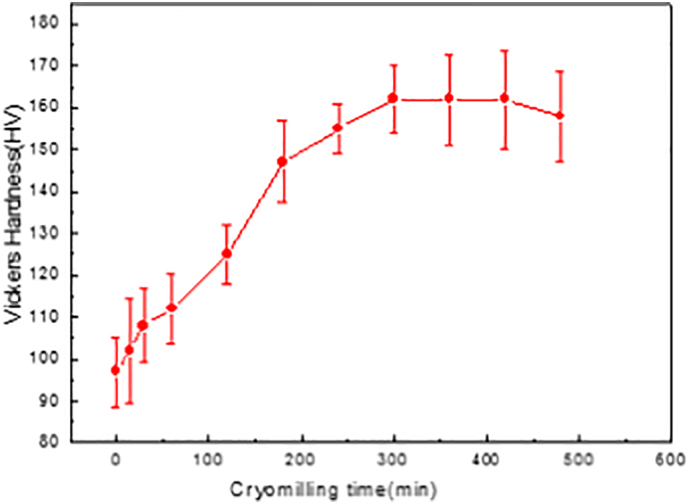
Microhardness results of cryomilled powders.

Fig. 2 presents the average Vickers microhardness results of all cryomilled powders. Due to grain refinement after cryomilling, the hardness is expected to increase. As shown in Fig. 2, the hardness increased substantially in the first 3 h. The rate of increase in hardness slowed down when the powder was further cryomilled to 4 and 5 h. It reached a plateau during cryomilling between 5 and 7 h.

For all the cryomilled powder, the main differences between them were particle size and corresponding internal grain size within particles. According to Hall-Petch strengthening theory [33], the hardness change behaviour in Fig. 2 could be related to the internal grain size evolution during cryomilling, which will be discussed in the following sections. As shown in Fig. 2, the highest average Vickers microhardness is 162 ± 10.7 HV after cryomilling for 5–7 h, which is by far the highest value reported for the Mg alloys compared to the recently reported highest hardness of 110.5 HV in a newly designed Mg alloy [34]. Finally, it slightly dropped after cryomilling for 8 h, although this change is not statistically significant.

### 3.3 Crystallite size measurement by XRD

The concept of a crystallite is different from a grain. A crystallite is the smallest un-faulted portion of the crystal while grains can be treated as the area within a polycrystalline material with the same crystallographic orientation and structure [35]. In some cases, grain size matches crystallite size obtained from TEM and XRD analysis, especially in nanocrystalline materials. The terminology crystallite is often used in XRD.

**Fig. 3:**
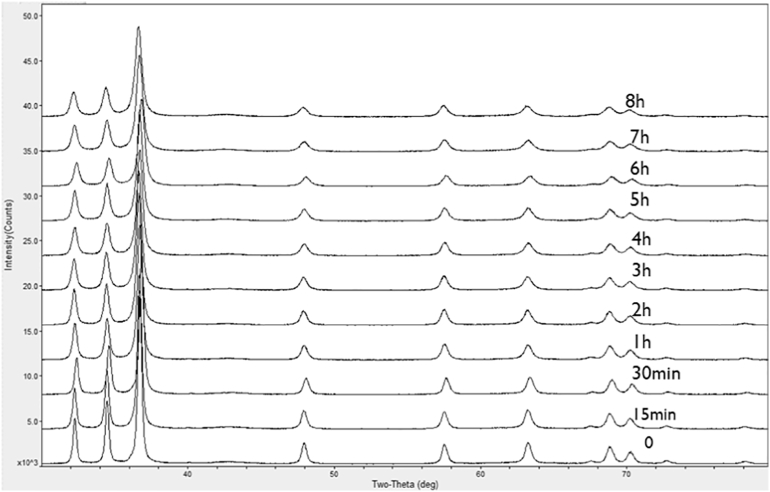
The XRD pattern of as-received and cryomilled Mg AZ31 powders.

**Fig. 4:**
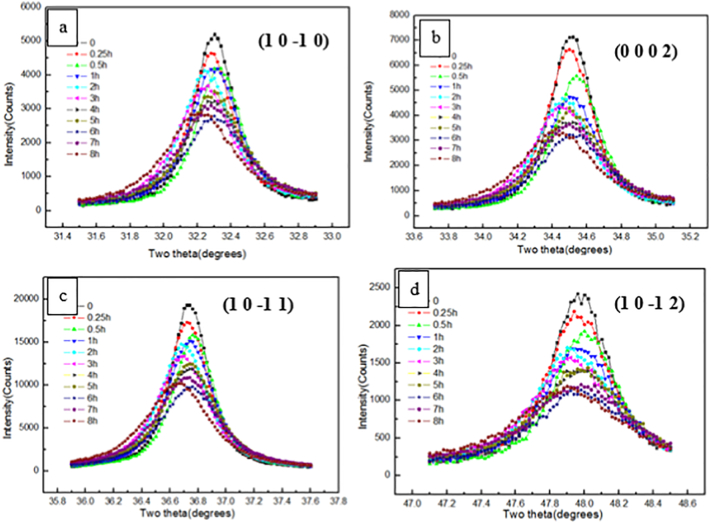
Four intensive peaks of the precursor and cryomilled Mg AZ31 powders.

Fig. 3 shows XRD patterns of precursor AZ31 and cryomilled powders. Compared to the precursor powder, significant peak broadening was evident after cryomilling for 4 h. To further observe the broadening effect, 4 high intensity peaks of magnesium were extracted and plotted in Fig. 4. Due to severe plastic deformation and very limited recovery and recrystallization during cryomilling, this peak broadening can be attributed to the ultra-fine crystallite size and high internal micro strain. Approximating the crystallite size and micro strain broadening profiles by a Cauchy function, the relationship between crystallite size (*d*) and the internal micro strain *ε* can be fitted using Williamson-Hull plot (eq. (1)) [36]:

**Figure:**
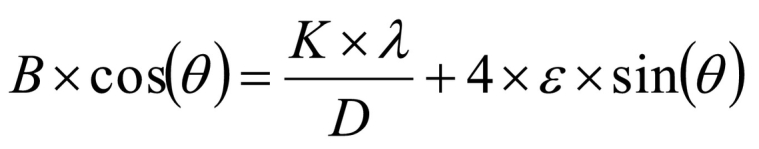

where *B* is the sample diffraction FWHM breadth, *θ* is the position of the peak maximum, *K* is a constant as 0.94, *λ* is the wavelength of the X-ray radiation (Cu, 0.154056 nm), *D* is the average crystallite size and *ε* is the micro strain. By performing a least squares fit to *B*cos(*θ*) against sin(*θ*) for all of the measured peaks of a sample, the average crystallite size was estimated to be 32 nm for AZ31 powder after 6–8 h cryomilling based on the intercept *Kλ/D*, as shown in Fig. 5, Fig. 6. The crystallite size was rapidly reduced in the first 3 h and the minimum crystallite size after cryomilling was 32 nm in this study. This was the reason why the hardness increasing rate was faster in the first 3 h while the increasing rate was slower after 3 h. The internal grain size evolution during cryomilling was also confirmed by the results of OM, SEM and TEM analysis below, which agreed with the crystallite size reducing trend measured by XRD.

**Fig. 5:**
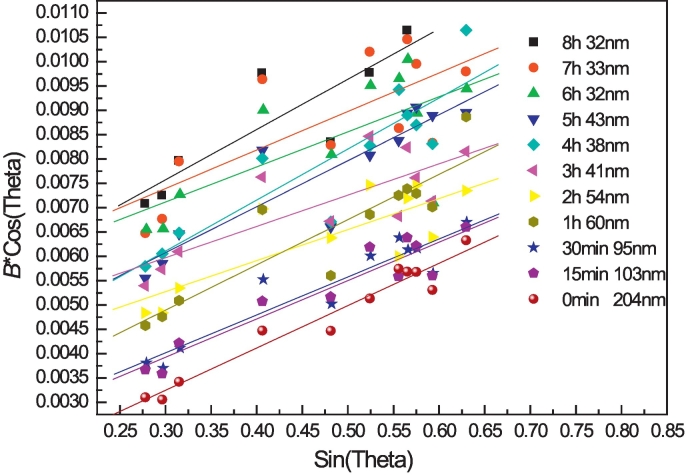
Crystallite size fitting curves of cryomilled powders at various cryomilling time.

**Fig. 6:**
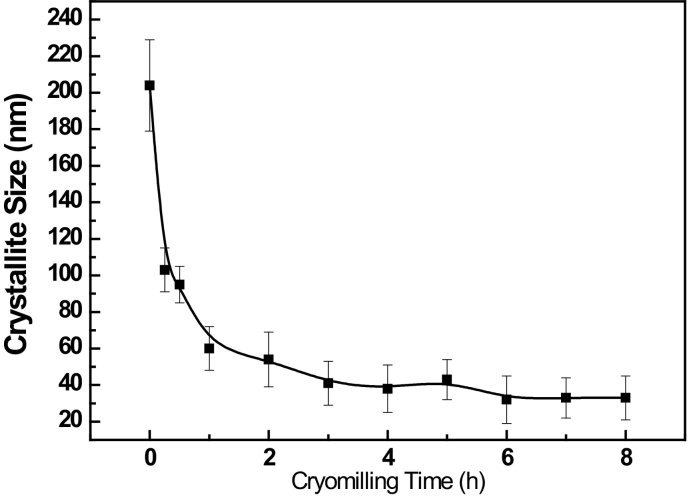
Crystallite sizes of cryomilled powders vs. cryomilling time.

### 3.4 Grain size measurement by a combination of OM, SEM and TEM techniques

**Fig. 7:**
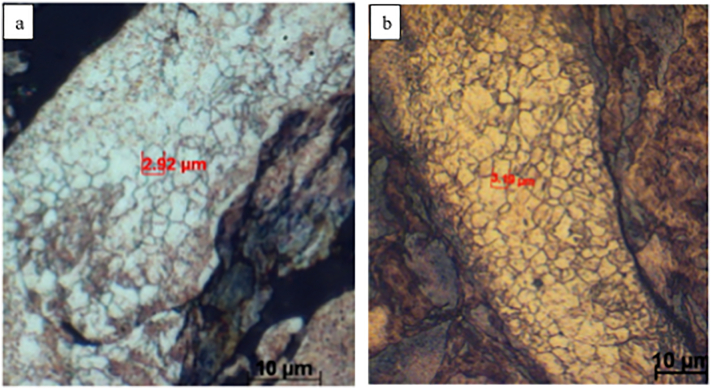
Typical OM images of etched cryomilled powders after (a)15 min, (b) 30 min.

**Fig. 8:**
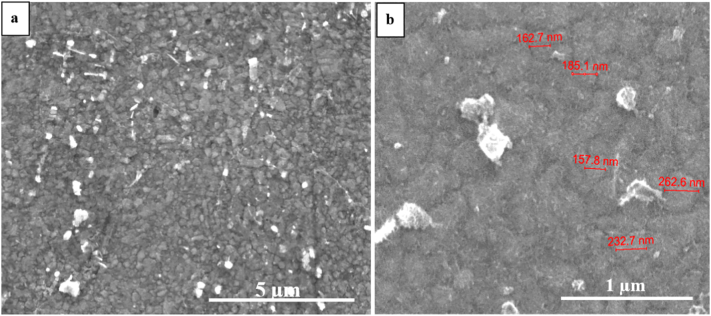
A secondary electron SEM image of Ultra-fine grains in a cyromilled (a)30 min and (b) 1 h particles.

Fig. 7 shows grain distributions for 15 min and 30 min cryomilled powders. The grain size in most of the particles was in the range of 1–5 μm and was not changed significantly compared to the precursor powder. The internal average grain size of the precursor powder was around 2 μm in our previous work [3]. However, very few particles of 30 min and 1 h cryomilled powder consisted of ultra-fine grains observed by SEM, as shown in Fig. 8, which indicated grain refinement started due to cryomilling. The size of some individual grains was measured and marked on the image when the powder was cryomilled for 1 h. Furthermore, the average grain size of 1 h cryomilled powder was calculated to be approximately 190 nm using linear intercept method.

Grains are expected to be further reduced with increasing cryomilling time. The resultant grain size exceeds the resolving power of the SEM and TEM was employed to observe these nanostructures. Because oxidation could not be avoided in the cryomilled Mg powders while preparing TEM samples, a very thin film of MgO film was always present on the surface and was responsible for additional diffraction ring pattern. To identify Mg and MgO diffraction patterns in the same diffraction pattern, a simulation of diffraction rings of Mg and MgO were plotted and used to distinguish MgO diffraction rings from Mg. Only 3 h, 6 h and 8 h cryomilled powders were selected to show the grain refinement process.

**Fig. 9:**
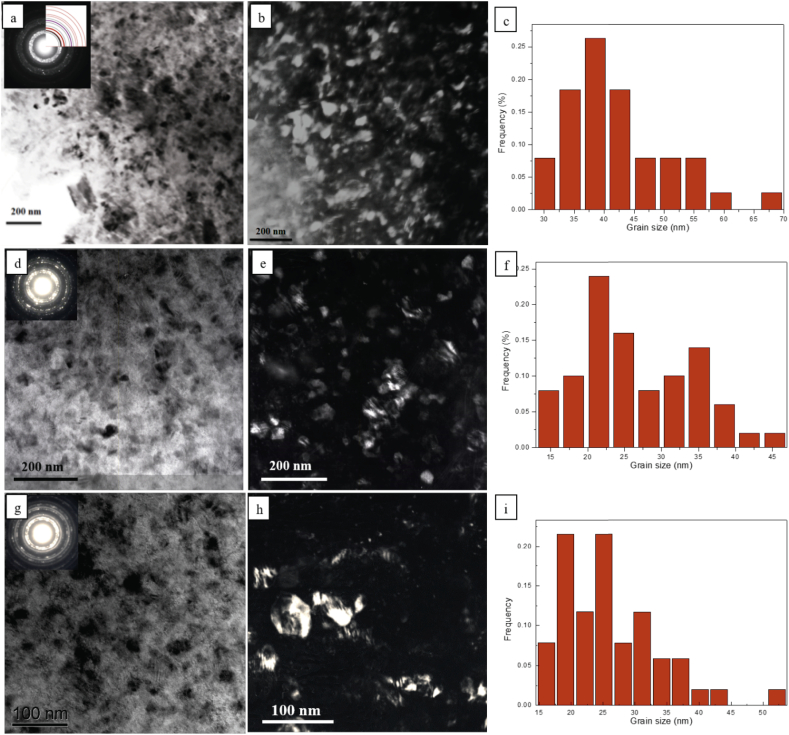
(a, d, g) Typical BF image and SAD pattern, (b, e, h) DF image and (c, f, i) grain size distribution histogram calculated from the TEM images of 3 h, 6 h and 8 h cryomilled powder, respectively.

Fig. 9(a) shows a selected area diffraction (SAD) pattern and BF image of the 3 h cryomilled powder. The red rings shown in Fig. 9(a) represent standard MgO diffraction rings while black rings correspond to Mg diffraction rings. The purple ring is the overlap diffraction ring of Mg (1 0 $\bar{1}$ 3) and MgO (220) crystal planes. The large number of diffraction spots, spread out in a complete ring for most planes indicated that most of grains were in the nano grain size regime with a random orientation. Fig. 9(b) is a dark field image, where fine crystallites can be clearly observed. The average grain size was measured to be 42.4 ± 12.2 nm based on well-defined grain boundaries. Fig. 9(c) shows the grain size distribution of the 3 h cryomilled powder (determined from 50 grains measured). It should be noted that very small grains were not included in determining the grain size distribution, since these small grains cannot effectively be distinguished from MgO dispersions. Fig. 9(d) shows TEM images of 6 h cryomilled powder. The BF and DF images exhibited a greater degree of fine microstructure was obtained after cryomillng for 6 h. The average grain size was further decreased. Fig. 9(f) displays the grain size distribution of the 6 h cryomilled powder (determined form 50 grains measure) and its average size is 26.2 ± 7.9 nm.

Fig. 9(g–h) presents TEM images of 8 h cryomilled powder. The BF and DF images showed that the grain size did not change significantly compared to the 6 h cryomilled powder 6 h. Fig. 9(i) shows the grain size distribution of the 8 h cryomilled powder (determined from 50 grains measure) and its average size is 26.1 ± 7.2 nm, which is very similar to that of 6 h cryomilled powder.

During the first three hours of cryomilling, the internal average grain size was significantly decreased from 1 to 5 μm to 42.4 nm. However, the grain size was only reduced from 42.4 nm after 3 h cryomilling to 26.1 nm after 8 h cryomilling. Therefore, the hardness increasing rate was much faster in the first 3 h of cryomilling than in the following 5 h, which agreed fairly well with the hardness results shown in Fig. 2.

Because the main difference between cryomilling and mechanical milling is the working temperature, the mechanism of nanostructure formation during cryomilling can be considered as a mechanical milling process [32]. Due to the cryogenic milling temperature, suppression of the recovery in the material was significant, which plays a positive role in reducing the milling time to obtain nanocrystallites [37]. Fecht [38] stated that there are three main stages of microstructural evolution during the mechanically milling process. Firstly, localized deformation occurs in shear bands consisting of high density dislocation arrays; secondly, because these dislocation arrays are not stable and recombination and annihilation of dislocations then ensue, this results in the formation of nano-sized subgrains; with further deformation, the subgrains with low angle grain boundaries (LAGB) transform to small grains with high angle grain boundaries (HAGB) by the annihilation and recombination of more dislocations into the boundaries or accompanying subgrains rotation during collision. Finally, this LAGB structure is able to change to a completely random HAGB between individual grains.

During the initial stage of cryomilling, the crystallite size measured by XRD was generally finer than corresponding grain size determined by OM, SEM and TEM methods. The reason for this is because XRD determines coherent diffraction domains including dislocation cells and subgrains [39]. After cryomilling for around 3 h, the crystallites size measured by XRD was similar to the grain size investigated by TEM. This indicated dislocations as well as subgrains started to transform into nano grains with HAGB, as shown by the diffraction rings patterns of cryomilled powders (Fig. 9).

The minimum grain size (d_min_) in this study was reached after 6 h based on crystallite size determined by XRD and TEM. No significant grain refinement occurred when further extending the cryomilling time. Furthermore, combining the results of particle size evolution and microhardness, it can be concluded that the shortest cryomilling time to obtain d_min_ should be only 6 h. Moreover, the highest hardness was up to 162 HV even after only 5 h cryomilling. In our previous study, the thermal stability of cryomilled powder was systematically analysed and the results indicated the cryomilled powder exhibited excellent thermal stability during annealing at 350–450 °C (0.67–0.78 T/T_M_) [40]. For instance at 450 °C, the nano grains grew from 26 nm to 37 nm in the first 5 min and grew to approximately 60 nm after 15 min. However, the grain growth was limited when the annealing time was increased to 60 min. The average grain size remained stable less than approximately 60 nm even after long anneals at temperatures as high as 450 °C. Therefore, it is promising that the cryomilled Mg AZ31 powder developed by short cryomilling with high hardness in this work can be used in high temperature applications where traditional Mg alloys are not used due to their poor heat-resistance properties [41].

### 3.5 Chemistry

Based on the investigations above, the precursor and 6 h cryomilled powders were chosen for chemical analysis to determine the composition changes after cryomilling, especially the impurity content. Results were listed in Table 2. It shows that the final chemistry of the powders was not changed significantly after 6 h cryomilling, particularly when compared to other studies relating to cryomilling [42,43]. Ertorer reported the content of N was increased from 0.017% to 2.03% and O from 0.19% to 0.263% after cryomilling [42]. Wen also stated the content of N and O in cryomilled powder was 0.26% and 0.512% respectively [43]. In general, powder contamination can be introduced either from the milling or handling environment. Because neither the process control agent (PCA) nor the liquid N_2_ slurry was mixed with the processed powder, N level was only slightly increased compared to other reported researches [42,43]. In contrast, the O level dropped to an undetectable level after cryomilling. For the precursor powder, there was a thick layer of MgO covering the particle surface, which was easy to be detected. However after cryomilling, the MgO film was completely broken-up and scattered uniformly as nano particles in the internal matrix, which resulted in the low detectable level of O level. In addition, Fe level was also not noticeably changed.

**Table 2:** Chemical analysis results for AZ31 powders (wt%).

Powder type	Mg	Al	Zn	Mn	N	O	Fe
As-received	96.15	3.02	0.80	<0.25	0.013	0.014	<0.25
CM6h	96.28	2.87	0.79	<0.25	0.060	<0.005	<0.25

## 4 Conclusions

In summary, nanostructured AZ31 powder was produced by cryomilling in this study. The particles underwent cold welding and fracture and its average particle size dropped to approximately 26 μm after cryomilling 6 h. The chemical analysis results indicated contamination introduced from cryomilling was not significant. The levels of contamination were small compared to other studies relating to cryomilling. The minimum average grain size of about 26 nm for the powder can be reached for a cryomilling time of 6 h or longer. The corresponding hardness of the 6 h cryomilled powder had the highest value of approximately 162 HV among all the cryomilled powders from 0.5 h to 8 h. Therefore, it can be concluded that 6 h cryomilling can reach the minimum grain size in this study based on the investigations of XRD and TEM. It also should be noted a few particles were agglomerated by other particles and cannot be processed by cryomilling due to cold welding.

## Data availability

The raw/processed data required to reproduce these findings cannot be shared at this time due to legal or ethical reasons.

## Declaration of Competing Interest

The authors declare that they have no known competing financial interests or personal relationships that could have appeared to influence the work reported in this paper.
